# Blastic plasmacytoid dendritic cell neoplasm: A case report

**DOI:** 10.3892/ol.2014.2836

**Published:** 2014-12-29

**Authors:** WEI WANG, WENSHENG LI, JIN-JING JIA, YAN ZHENG, HAO WANG, XIAO-MIN GAO, XIN-YU DONG, QIONG TIAN, HUI-LING JING, XIN WANG, SHENG-XIANG XIAO

**Affiliations:** 1 Department of Dermatology, Second Affiliated Hospital of Xi’an Jiaotong University, Xi’an, Shaanxi, P.R. China; 2 Department of Pathology, Third Affiliated Hospital of Xi’an Jiaotong University, Xi’an, Shaanxi, P.R. China

**Keywords:** blastic plasmacytoid dendritic cell neoplasm, cyclophosphamide, doxorubicin, vincristine and prednisolone chemotherapy, prognosis, treatment, diagnosis

## Abstract

Blastic plasmacytoid dendritic cell neoplasm (BPDCN), formerly known as agranular cluster of differentiation (CD)4^+^/CD56^+^ hematodermic neoplasm, is a rare and aggressive type of lymphoma, with only ~100 cases reported worldwide. BPDCN is a hematological malignancy derived from precursors of plasmacytoid dendritic cells and is clinically characterized by cutaneous manifestations involving the lymph nodes and peripheral blood, a leukemia-like dissemination and a poor prognosis. The present study reports the case of a 54-year-old male who presented with symptoms characteristic of BPDCN. Pathological and immunohistochemical analysis of abdominal skin lesion biopsies were used to determine a diagnosis of stage IIIE BPDCN. Although cyclophosphamide, doxorubicin, vincristine and prednisolone chemotherapy was administered, the patient succumbed to BPDCN nine days after the discontinuation of chemotherapy. Thus, the period from BPDCN presentation to mortality was ≤3 months. The case reported in the present study was characterized by rapid development and poor prognosis, and displayed additional features of BPDCN, including systemic dissemination and a short survival period.

## Introduction

Blastic plasmacytoid dendritic cell neoplasm (BPDCN) is a rare and highly invasive type of malignant hematopoietic and lymphoid tissue tumor (1). Since BPDCN was initially reported by Adachi *et al* (2) in 1994, it has been successively reported in the literature. In 2004, Chaperot *et al* (3) identified that the BPDCN tumor functions similarly to plasmacytoid dendritic cells (pDC), and, thus, proposed that it may be derived from the precursor of the pDC. Subsequent studies determined that the BPDCN tumor cells express the highly specific pDC markers, blood dendritic cell antigen (BDCA)-2/cluster of differentiation (CD)303 and BDCA-4/CD304, supporting the hypothesis that the BPDCN tumor is derived from pDC (4). In 2005, the World Health Organisation (WHO) European Organization for Research and Treatment of Cancer classification of cutaneous lymphomas recommended the use of the term CD4^+^/CD56^+^ hematodermic neoplasm (5,6). In 2008, the tumor was officially named BPDCN in the WHO classification of lymphoid and hematopoietic tumors and was listed as a novel, independent type of hematopoietic and lymphoid tissue disease (7).

BPDCN can occur in individuals of all ages (range, 8 months-103 years), however, it predominantly occurs in the elderly (8). Skin involvement is the most prominent clinical feature and includes isolated, confined or generalized plaques or nodules. The plaque diameter ranges from a few millimeters to over ten centimeters, while the color ranges from dark red to characteristic purple, and ulcers occasionally occur. The manifestations of this disease also occasionally involve the mucosae (9,10). In addition to the initial manifestation of skin lesions, the disease involves other systems. For example, lymphadenectasis occurs in 40–50% of patients, the bone marrow and peripheral blood are involved in 60–90% of patients, and splenomegaly occurs in just 20% of patients (1). The central nervous system is rarely involved during the early stages of disease onset, however, it is frequently involved in cases of disease recurrence. Group B symptoms, such as fever, night sweats and weight loss, seldom occur. Auxiliary examination often identifies pancytopenia and most commonly, thrombocytopenia. Furthermore, leukemia is a common feature in the end stage of progressive or recurrent cases, and 10–20% of BPDCN cases are accompanied by, or can progress to, acute myeloid leukemia (11).

## Case report

**Figure 1:**
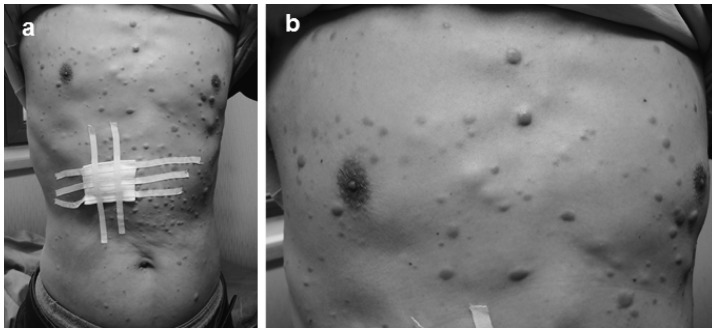
Systemic multiple nodules and plaques on the (a) trunk and (b) chest of the patient.

On 30th January, 2013, a 54-year-old male presented to the Department of Dermatology, Second Affiliated Hospital of Xi’an Jiaotong University (Xi’an, China) with systemic multiple nodules and lumps accompanied by pain in the limbs. Physical examination did not identify any evident abnormalities of the heart, lungs or abdomen, however, dozens of multiple papules, plaques and subcutaneous nodules were identified, predominantly distributed on the trunk (Fig. 1), with a few scattered on the head and limbs. The characteristics of these plaques and nodules were as follows: Different textures (hard, tough, subcutaneously located or protruding out of the skin); different sizes (diameter range, 0.5–5.0 cm); poor motility (with the largest on the left shoulder); varying colors (pale pink, skin color or prunosus); smooth surface with no scales; and a certain degree of pain caused by applying pressure. Furthermore, the hair, mucous membranes, fingernails and toenails of the patient appeared normal. Lymph nodes (size, 1–2 cm) on the left armpit, and each side of the neck and groin were palpable. These nodes were rough, hard and were not painful.

**Figure 2:**
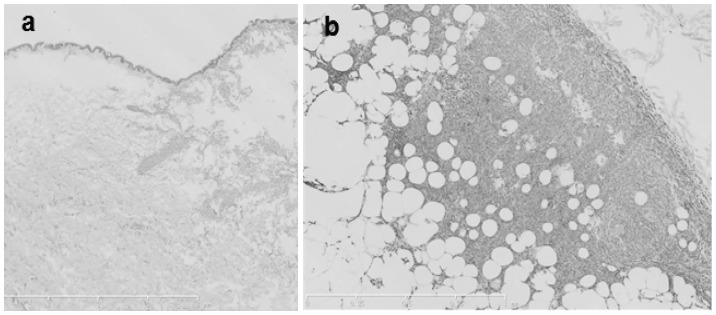
Pathological examination of the abdominal skin lesions, demonstrating (a) no involvement of the epidermis (scale bar, 4 mm), and (b) dermis and subcutaneous fat layer infiltration by medium-sized tumor cells (sale bar, 1 mm). Staining, hematoxylin and eosin.

**Figure 3:**
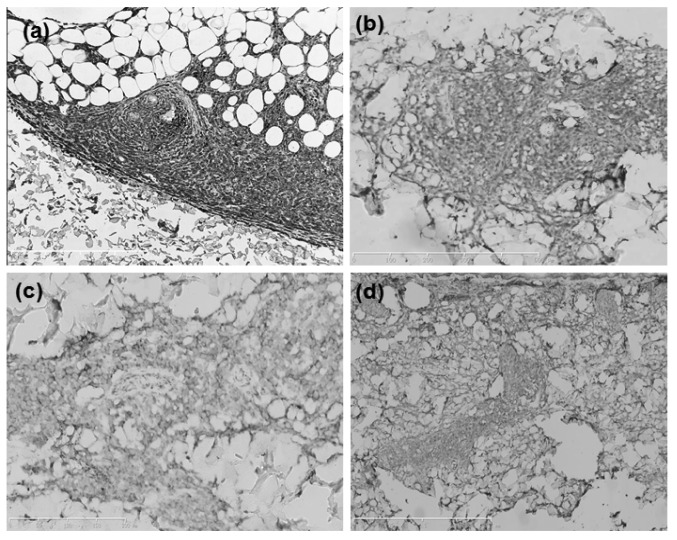
Immunohistochemical analysis of the abdominal skin lesions determined that the tumor cells were positive for (a) leukocyte common antigen (scale bar, 1 mm), (b) cluster of differentiation (CD)4 (scale bar, 500 μm), (c) CD56 (scale bar, 200 μm) and (d) CD43 (scale bar, 1 mm).

**Figure 4:**
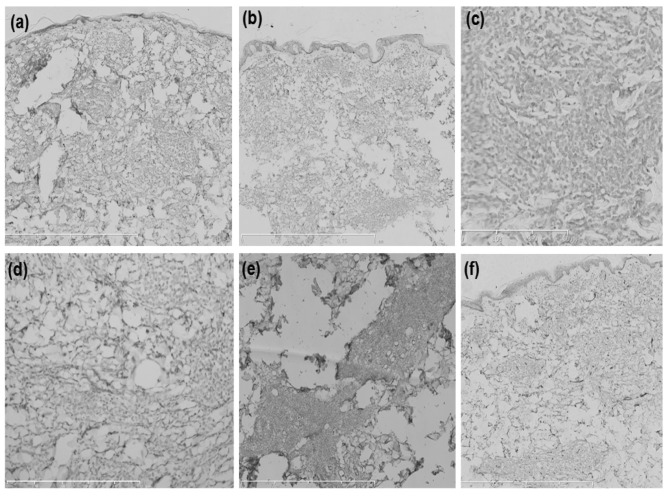
Immunohistochemical analysis of the abdominal skin lesions determined that the tumor cells were negative for (a) cluster of differentiation (CD)3 (scale bar, 1 mm), (b) CD7 (scale bar, 1 mm), (c) CD8 (scale bar, 300 μm), (d) CD20 (scale bar, 500 μm), (e) CD30 (scale bar, 500 μm) and (f) CD34 (scale bar, 1 mm).

**Figure 5:**
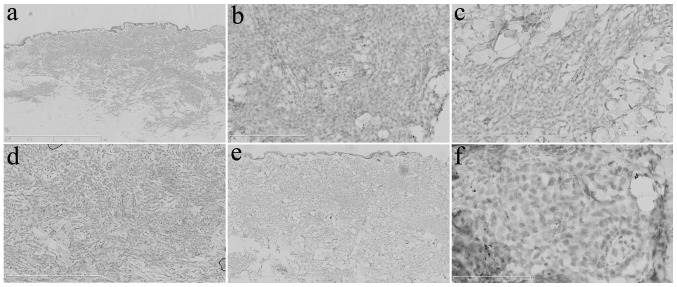
Immunohistochemical analysis of the abdominal skin lesions determined that the tumor cells were negative for (a) cluster of differentiation (CD)68 (scale bar, 2 mm), (b) CD123 (scale bar, 200 μm), (c) myeloperoxidase (scale bar, 300 μm), (d) Epstein-Barr virus-encoded RNA (scale bar, 500 μm), (e) terminal deoxynucleotidyl transferase (scale bar, 1 mm) and (f) PAX-5 (scale bar, 100 μm).

Data from a routine blood and liver kidney function test were normal, however, a bone marrow biopsy demonstrated active hyperplasia, 27% promyelocytic leukemia cells and positive leukocyte peroxidase (myeloperoxidase) staining. Additionally, pathological examination of the abdominal skin lesions identified that the epidermis was not involved, however, the dermis and subcutaneous fat layer were infiltrated with diffused and dense medium-sized tumor cells (Fig. 2). The characteristics of these tumor cells were as follows: All cells were of a similar size and lymphoblast-like form; the chromatin was fine and smooth; the nucleoli were prominent, with a round or oval shape; insignificant vascular proliferation occurred, with no vascular invasion or necrosis; and minimal inflammatory cell infiltration was observed. Immunohistochemical staining with leukocyte common antigen (LCA), CD4, CD56 and CD43 was positive (Fig. 3), however, staining with CD3, CD7, CD8, CD20, CD30, CD34, CD68 (Fig. 4) CD123, myeloperoxidase, Epstein-Barr virus-encoded RNA (EBER) and terminal deoxynucleotidyl transferase (TdT) and PAX-5 was negative (Fig. 5), with a Ki67 labeling index of 40–50%. These findings fulfilled the requirements for the diagnosis of BPDCN as stage IIIE (12–14). Relevant examinations did not demonstrate any chemotherapy contraindications, thus, a cyclophosphamide, doxorubicin, vincristine and prednisone (CHOP) chemotherapy program [750 mg/m^2^ intravenous (i.v.) cyclophosphamide, 1st day; 50 mg/m^2^ i.v. doxorubicin, 1st day; 1.4 mg/m^2^ i.v. vincristine, 1st day; 100 mg/m^2^ oral prednisone, every day, 1st–5th day]was initiated. Following the first course of chemotherapy, the nodules significantly decreased in size, however, the patient developed fever, pharyngalgia and hoarseness. Despite the administration of an antibiotic treatment (2.0 g ceftriaxone, twice a day for 10 days), the patient developed sepsis, septic shock, metabolic acidosis, respiratory acidosis, hypokalemia, hyponatremia, hypochloremia, hypocalcemia, hypoxemia, cardiac insufficiency, bone marrow inhibition, agranulocytosis and extreme thrombocytopenia. The patient succumbed nine days after the first course of chemotherapy had ended.

## Discussion

The present study describes a case of BPDCN presenting with skin lesions. The case presented with an initial typical manifestation of skin involvement, accompanied by lymph node and bone marrow involvement. The skin tissue pathology was typical of BPDCN, and immunohistochemistry demonstrated positive LCA, CD4, CD56 and CD43 staining, and excluded a myeloid and B or T cell lineage and origin. Furthermore, immunohistochemistry demonstrated that the tumor was EBER-negative, indicating that the disease was not associated with EB virus infection. Thus, the disease was eventually diagnosed as BPDCN (stage IIIE). The unique aspect of the present case was that the pDC-specific marker, CD123, was negative. The patient experienced rapid disease progression. Due to personal reasons, the patient did not undergo allogeneic hematopoietic stem cell transplantation, instead, CHOP chemotherapy was administered. However, this program exhibited a poor curative effect and the patient succumbed nine days after the first course of chemotherapy ended.

BPDCN is a rare form of lymphoma-like disease (1). The pathogenesis of BPDCN remains unclear. Wiesner *et al* (15) investigated histopathological skin lesion specimens of 14 BPDCN patients and demonstrated that chromosomes 9, 12, 13 and 15 were more likely to be absent, and that the cyclin dependent kinase inhibitor (CDKN) 1B site was most commonly not present (detectable in 64% of the tumors). Furthermore, CDKN2A-alternative reading frame-CDKN2B sites occurred in 50% of the cases. These results indicate that the mutation of the key cell cycle regulatory proteins p27, p16 and retinoblastoma 1 may be important in the change of the degree of malignancy in BPDCN (15).

BPDCN has a unique immunophenotype, as it is CD4^+^ and CD56^+^, and is generally CD43^+^. CD7 and CD2 are expressed in varying degrees, and in approximately half of all cases, tumor cells are CD68^+^ and exhibit a specific cytoplasmic granular shape (i.e., multifocal color imaging of the Golgi apparatus). Certain cases are TdT^+^ (range, ~10–80%), while S-100 protein positivity has also been demonstrated (16). Additionally, BPDCN tumor cells often express the pDC-specific surface marker CD123. T cell leukemia/lymphoma 1, CD2-associated protein, BDCA-2/CD303, BDCA-4/CD304 and other pDC-associated antigens all increase the basis for diagnosis (17,18). CD123 is the α chain of the interleukin-3 receptor, and is a sensitive and specific marker of pDC (7,17,18). As it has been demonstrated that BPDCN cells are derived from pDCs, certain studies have proposed that CD123 may be a specific marker for the diagnosis of BPDCN (19–22). However, CD123 is not expressed in all BPDCNs (23) and can be highly expressed in normal granulocytes, as well as in acute granulocytic leukemia, histiocytosarcoma and Langerhans cell histiocytosis psychosis.

A diagnosis of BPDCN is typically determined based on histopathological and immunohistochemical examinations. In general, BPDCN can only be diagnosed when the tumor cells demonstrate a blastic morphology, a CD4^+^/CD56^+^ immunophenotype, and no myeloid and T or B cell-specific surface marker expression. Additionally, these immunohistochemical properties are used to distinguish between BPDCN and other skin diseases caused by leukemia. However, it is important to be aware of the atypical pathological manifestations of BPDCN, such as tumor cell invasion solely around blood vessels or appendages and the polymorphic tumor cells (24).

BPDCN is characterized by high aggressiveness, rapid progression and a poor prognosis, with patients exhibiting a median survival time of 12–14 months. The survival time may be associated with the tumor stage and the age and clinical manifestations of the patient (25,26).

The present case was a typical case, whereby skin involvement was the first manifestation, with lymph node and bone marrow involvement. There is a typical manifestation of BPDCN in histopathology, and immunohistochemical staining showed LCA, CD4, CD56 and CD43 positivity, excluding B, T and myeloid cell lineage and origin. EBER staining was negative, which indicated that there was no association with EB virus infection. The final diagnosis was BPDCN (stage IIIE) and, notably, CD123 was negative in this case, which was considered as a unique cell marker for BPDCN. Due to personal reasons, the patient did not receive allogeneic hematopoitic stem cell transplantation, and instead received CHOP chemotherapy. However, the treatment efficacy was poor and the patient succumbed nine days after the first course of chemotherapy had ended. In summary, BPDCN is a rare type of lymphatic and hematopoietic tumor, the current understanding and effective treatment of which remain to be further investigated.
